# Intramuscular hemangioma of the masseter muscle: a case report

**DOI:** 10.1186/1757-1626-2-7459

**Published:** 2009-05-18

**Authors:** C D Narayanan, Preeth Prakash, C K Dhanasekaran

**Affiliations:** 1 Department of Surgery, Sri Ramachandra UniversityPorur, Chennai 600116India; 2 Department of Surgery, Sri Ramachandra UniversityPorur, Chennai 600116India; 3 Department of Oral and Maxillofacial Surgery, Sri Kumaran Hospital1, Link Road, Kottur Gardens, Chennai 600085India

## Abstract

Intramuscular hemangiomas are uncommon neoplasm's arising most frequently in the masseter and trapezius muscle. Due to it's location it is often mistaken for a parotid swelling and rarely is an accurate pre-operative diagnosis achieved clinically. The intra masseteric location also poses special problem in terms of proximity to the facial nerve and the post operative flattening following excision of the masseter muscle. A case of intramuscular hemangioma in a 17 year old girl is presented. Inadequacy of computed tomography scan and cytology in achieving a pre-operative diagnosis and also the treatment modalities are reviewed here. An estrogen receptor and progesterone receptor study has been done to verify the hormonal basis of this tumour.

## Case presentation

A 17 year old girl from India, Asia presented with a swelling in the right cheek of five months duration. She first noticed the swelling while washing her face. Swelling gradually increased in size, becoming more pronounced during mastication and while waking up in morning. Over the last one month she developed pain over the swelling. She gave no history of trauma or oral contraceptive pill usage. On physical examination there was a swelling in the region of the right parotid measuring 3 x 2 cm which was non-tender. Swelling was 3 cm in front of the tragus and 2 cm below the zygoma. There was no compressibility and the overlying skin was normal. On clenching the masseter the swelling diminished in size. There was no facial nerve involvement and parotid duct orifice was normal.

**Figure 1.**
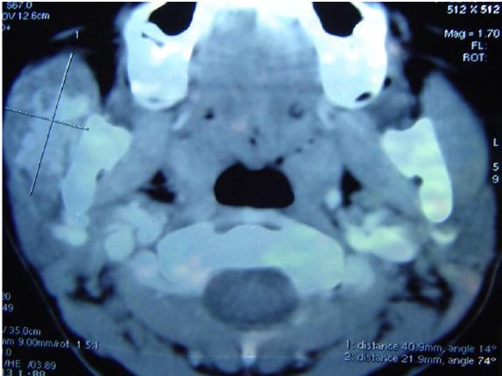
Contrast Computed Tomography (CT). Contrast CT film shows the heterogenous vascular lesion involving the right masseter.

**Figure 2.**
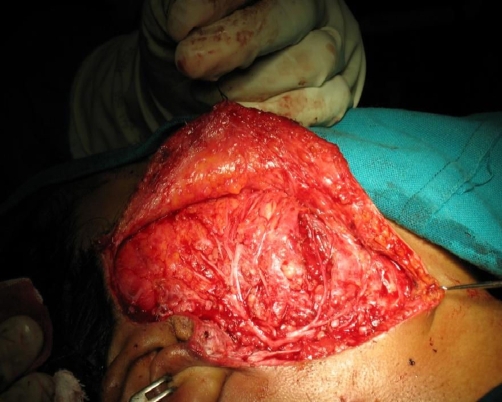
Facial nerve after completion of superficial parotidectomy. The branches of the facial nerve have been dissected after completion of superficial parotidectomy. The nerve is splayed over the vascular neoplasm involving the masseter.

**Figure 3.**
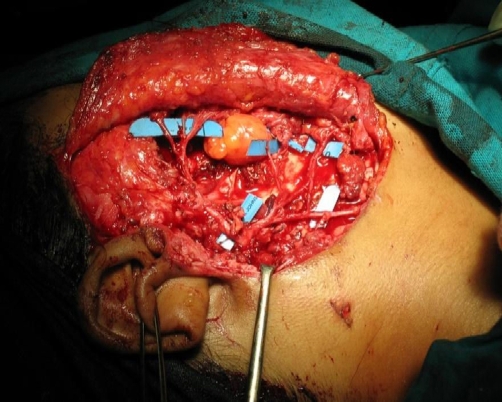
Intra-operative view following completion of dissection. The masseter along with the hemangioma has been excised from the zygomatic arch to the lower border of the mandible. The facial nerve branches have been preserved and the buccal pad of fat lies exposed.

FNAC revealed greenish color aspirate, cytology of which did not reveal any cellular material, this probably was extravasated blood and the greenish tinge, due to breakdown products of hemoglobin. This was evident as areas of hemorrhage on histology. A contrast CT was done (Figure 1) which showed a well defined heterogenous mass lesion involving the right masseter muscle which was highly vascular and a diagnosis of rhabdomyosarcoma was made. Preauricular skin incision was made as for a parotidectomy. Skin flaps were raised A normal looking parotid was found and the underlying masseter showed a diffuse bulge with no surface abnormality. The facial nerve trunk was identified and a superficial parotidectomy was done after carefully identifying and preserving the branches of the facial nerve. The facial nerve was seen spread over the diffuse bulge involving the masseter muscle (Figure. 2). The nerve fibres were subsequently dissected from its masseteric bed and gently raised with a hook. There was no definite encapsulated lesion or a palpable swelling which could be excised. The whole masseter exhibited compressibility with gradual filling. The facial nerve branches were gently lifted with a wooden spatula and under this arch the masseter was mobilized from the mandible and also severing it's attachment from the zygomatic arch. Through the same incision the external carotid artery was slinged and proximal vascular control was achieved. Anteriorly the masseteric fascia was preserved protecting the fine communications of the facial nerve. (Figure 3) displays the branches of the nerve after excision of the masseter. Complete excision of the masseter did not cause any significant morbidity in terms of cosmesis. There was temporary paresis of the marginal mandibular nerve which recovered in 4 weeks. The histopathological examination revealed capillary hemangioma. An ER [Estrogen Receptor] and PR [Progesterone Receptor] study was done which was negative.

## Discussion

Hemangiomas of skeletal muscle represent 0.8% of all benign vascular neoplasm [1]. Of these 13.8% occur in the head and neck region [2], with the masseter muscle being the most common site, followed by the trapezius and sternocleidomastoid muscles respectively [2,3]. Intramuscular Hemangiomas [IMH] generally occur in the first three decades of life [4]. Although intramuscular hemangiomas have shown an equal sex distribution, involvement of the masseter has a definite male predominance [5].Various theories have been proposed to explain its etiology. The congenital nature is supported by the fact that it usually presents in the first three decades of life [3,6,7]. Others have suggested it arises from malformed tissue subjected to repeated trauma [3]. Many have speculated a possible hormonal role on the growth of IMH as there was sudden increase in size noted on taking OC pills [1,2,5,8,9]. However our studies on ER & PR were negative.

Allen & Enzinger classified them as large vessel [>140 mm in diameter] small vessel [<140 mm in diameter] and mixed vessel types [10]. They correspond to cavernous, capillary, and mixed type respectively. This classification is useful and correlates well with clinical presentation and recurrence rates. The capillary type of hemangioma occurred more frequently in the head and neck region. The highly cellular nature of many capillary hemangiomas may explain the lack of clinical signs usually associated with vascular lesions, thus rendering pre-op diagnosis difficult. The cavernous and mixed types occurred more frequently in the trunk and lower limbs. The mixed type had the greatest tendency for local recurrence [28%].

These tumours present as gradually enlarging mass lesions with duration often less than a year [2]. Accurate preoperative diagnosis has been reported in less than 8% of cases in view of its intramuscular location and the overlying parotid. Bruits, thrills, compressibility are often absent unlike in other vascular malformations [2]. The most common clinical presentation is a mass with associated pain symptoms in 50 to 60% of cases. There are usually no skin changes. Clenching the teeth could make the lesion to become more firm and fixed.

A variety of tumours can be confused clinically with an IMH. Most of them are often mistaken for salivary neoplasms & the differential diagnosis include cysts, lymphangiomas, rhabdomyosarcomas, masseteric hypertrophy, and schwannomas.

FNAC is inconclusive in arriving at a diagnosis as it yields only a bloody aspirate [4]. Superselective arteriography with subtraction clearly defines the altered vascular pattern and flow dynamics including feeder vessels and also opens up therapeutic modalities. However it may fail to demonstrate low flow lesions adding to the diagnostic difficulty.

Though contrast CT may demonstrate the vascular nature of the tumour MRI has shown superiority in the exquisite delineation and contrast of the lesion from it's surrounding due to its multiplanar capability.

The management of IMH should be individualized based on such factors as tumour location, age, depth of invasion, Cosmesis. Many treatment modalities like cryotherapy, radiation therapy, steroid administration and embolization have been advocated but the treatment of choice at present remains surgical excision [1,2,9]. Local recurrence ranging from 9 to 28% have been reported even after wide excision [2], hence we recommend that total excision of the masseter ensures that there is no recurrence. This is associated with very little cosmetic and functional disability. Difficulty in intraoperative localization of the exact extent of the tumour due to its supple nature and the absence of a definite capsule justifies a complete excision of the masseter. The fibrosis following surgery may render reexploration and excision in case of recurrence hazardous with more risk of damage to the facial nerve.

The preauricular incision combined with a superficial parotidectomy allows for a complete excision with preservation of branches of the facial nerve with very little cosmetic and functional disability. Intraoral approaches give limited exposure to the facial nerve branches and often result in nerve injury [2].

Any lesion in the region of the parotid must be evaluated thoroughly prior to surgery. If the FNAC shows a bloody aspirate on repeat sessions, the possibility of a vascular lesion must be thought of. MRI should be the next line of investigation. Arteriography is usually not necessary. As proximal vascular control can be achieved through the same incision, total masseteric excision has very little cosmetic or functional disability and offers a better chance of clearance.

**Figure 4.**
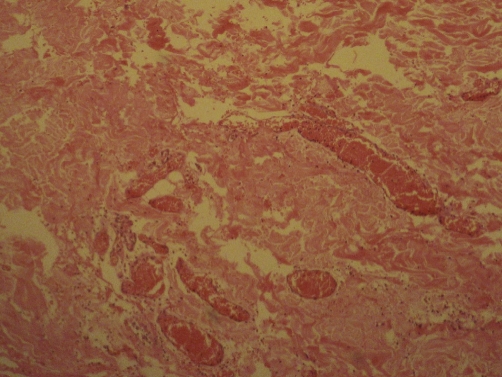
Histopathological view of the lesion. Section shows skeletal muscle fibres infiltrated by angiomatous lesion composed of thin walled vascular channels lined by single layer of endothelium, suggestive of intramuscular hemangioma. Much extravasated hemorrhage is seen.

## List of abbreviations

CT: Computer Tomography
ER: Estrogen Receptor
FNAC: Fine needle aspiration cytology
IMH: Intramuscular Hemangiomas
MRI: Magnetic resonance imaging
PR: Progesterone Receptor
